# Vaccination Drives Changes in Metabolic and Virulence Profiles of *Streptococcus pneumoniae*

**DOI:** 10.1371/journal.ppat.1005034

**Published:** 2015-07-16

**Authors:** Eleanor R. Watkins, Bridget S. Penman, José Lourenço, Caroline O. Buckee, Martin C. J. Maiden, Sunetra Gupta

**Affiliations:** 1 Department of Zoology, University of Oxford, Oxford, United Kingdom; 2 Center for Communicable Disease Dynamics, Department of Epidemiology, Harvard School of Public Health, Boston, Massachusetts, United States of America; Emory University, UNITED STATES

## Abstract

The bacterial pathogen, *Streptococcus pneumoniae* (the pneumococcus), is a leading cause of life-threatening illness and death worldwide. Available conjugate vaccines target only a small subset (up to 13) of >90 known capsular serotypes of *S*. *pneumoniae* and, since their introduction, increases in non-vaccine serotypes have been recorded in several countries: a phenomenon termed Vaccine Induced Serotype Replacement (VISR). Here, using a combination of mathematical modelling and whole genome analysis, we show that targeting particular serotypes through vaccination can also cause their metabolic and virulence-associated components to transfer through recombination to non-vaccine serotypes: a phenomenon we term Vaccine-Induced Metabolic Shift (VIMS). Our results provide a novel explanation for changes observed in the population structure of the pneumococcus following vaccination, and have important implications for strain-targeted vaccination in a range of infectious disease systems.

## Author Summary

The bacterium *Streptococcus pneumoniae* is a major cause of life-threatening pneumonia, septicaemia and meningitis worldwide. Pneumococci are covered by a polysaccharide capsule of which there are over 90 distinct serotypes. Available vaccines target a small subset (either 7, 10 or 13) of these capsular serotypes but, following their introduction, increases in the relative amount of disease caused by non-vaccine serotypes have been observed in several countries. Here we offer an alternative explanation for this phenomenon to the traditional concept of Vaccine-Induced-Strain-Replacement whereby the removal of interference from vaccine strains allows non-vaccine strains to fill the niches left vacant by them. We show, instead, that vaccination induces genotypic changes among non-vaccine strains which can lead to an increase in both transmissibility and virulence. Using a mathematical model of genomic evolution, in which strains are split into antigenic, metabolic and virulence-associated components, we show that metabolic and virulence-associated components originally associated with vaccine serotypes become associated with non-vaccine serotypes following vaccination. We term this Vaccine-Induced-Metabolic-Shift and propose that it explains post-vaccine changes observed in pneumococcal population structure in a number of locations worldwide.

## Introduction

**Fig 1:**
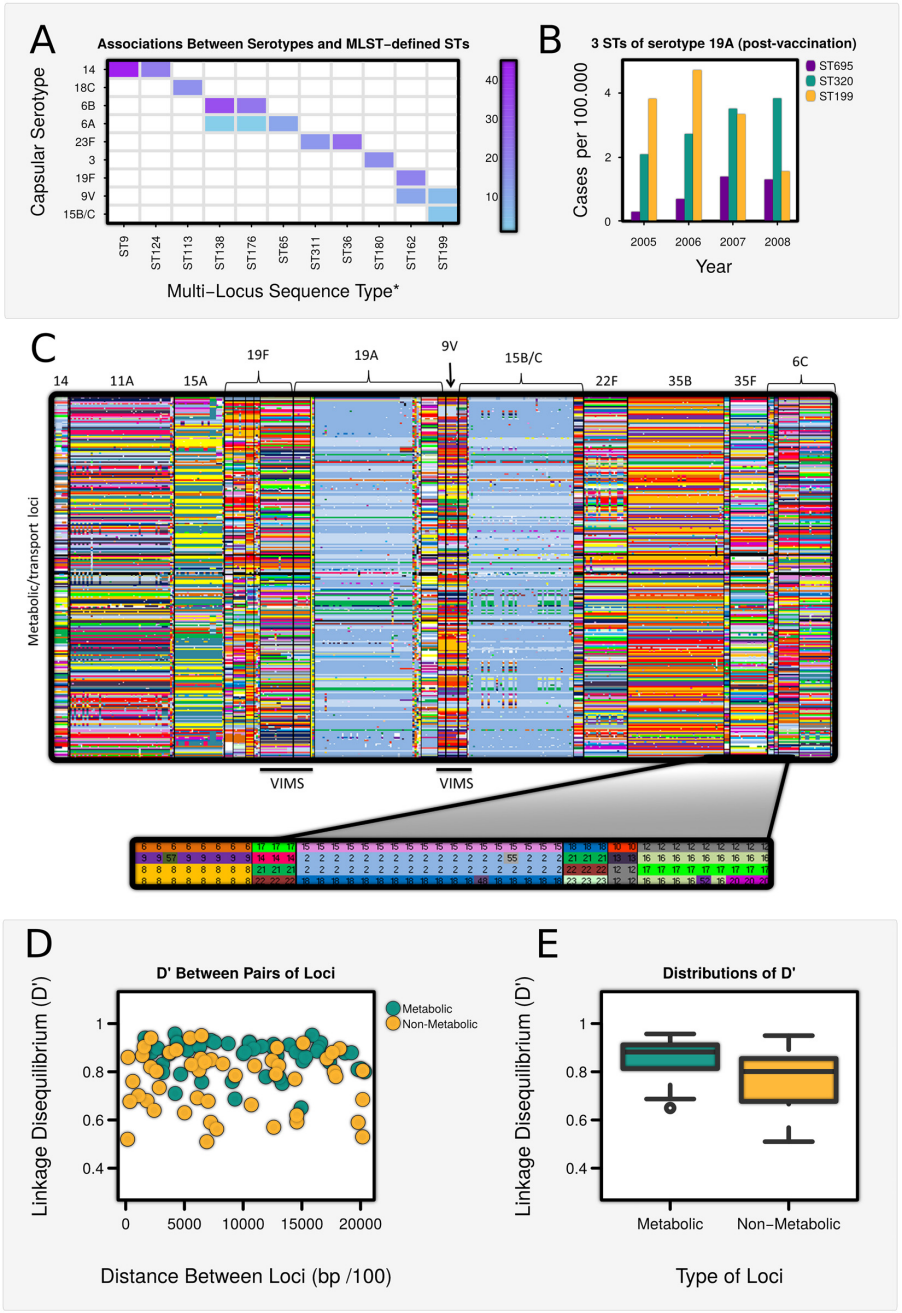
Shifts in associations between capsular serotype and metabolic types type following vaccination. (**A**) Associations between the most frequent pneumococcal serotypes and MLST-defined STs, England (adapted from [4]) (**B**) Disease prevalence of three STs of serotype 19A among children <5 years in the United States, following vaccination in 2000 (adapted from [7]) (C) Partial screenshot of the metabolic profiles of 616 pneumococcal isolates (obtained from [22]) grouped by serotype (see also S1 Dataset). Each column represents the allelic profile of 876 metabolic/transport genes of a single isolate. Black bars indicate where VIMS (Vaccine-Induced Metabolic Shift) has occurred. (D) Linkage Disequilbrium (measured as D’) between pairs of metabolic loci & pairs of non-metabolic loci, plotted against their genetic distance on the chromosome. (E) Box-plots showing the distribution of D’ among metabolic and non-metabolic pairs of loci. Values of D’ were significantly higher among metabolic/transport loci than non-metabolic loci (Wilcoxon Test: W = 615, p<0.0001).

Pneumococci can be stratified into over 90 different “serotypes” according to the antigenic properties of their polysaccharide capsule; only a small number (~10) of these, however, are responsible for most cases of invasive disease worldwide [1]. Pneumococcal populations are also highly diverse in non-antigenic genes, and are commonly classified into sequence types (ST) by Multi Locus Sequence Typing (MLST) of seven metabolic housekeeping genes [2]. Given the high rates of recombination observed in the pneumococcus [3], it might be expected that most serotypes would be linked to a variety of STs, yet many studies (eg [4]) show an intriguing pattern of largely non-overlapping associations between capsular serotype and ST in pneumococcal populations (Table A in S1 Text and Fig 1A). These associations are not stable: capsular switching events (whereby an ST acquires a different capsular serotype) have been documented to occur regularly throughout the past 7 decades [5], and since the introduction of the heptavalent PCV7 vaccine (first licensed in the USA in 2000), it has been noted that many STs that were previously associated with vaccine serotypes now occur in association with non-vaccine serotypes. For example, ST320 (previously associated with the vaccine serotype 19F [6]) has replaced ST199 as the most common MLST type associated with non-vaccine serotype 19A in the US [7–10] (Fig 1B). Increases have also occurred in the prevalence of the ST695^19A^ strain in which the vaccine serotype 4 capsule has been switched for a 19A capsule [11] (Fig 1B). Similarly, in Korea, where PCV7 caused a drop in vaccine serotypes 23F and 19F (but notably not 6B), ST81 (previously associated with serotypes 23F and 19F) is now the primary MLST type of serotype 6A [12]. Several theoretical models have demonstrated the potential of strain-targeted vaccines to increase the prevalence of non-vaccine serotypes due to the removal of cross-immunity or direct resource competition [13–19], but these do not explain why changes have occurred in the MLST composition of non-vaccine serotypes.

We have previously proposed that a combination of immune-mediated interference between identical antigenic types and direct competition between identical metabolic types can generate non-overlapping associations between antigenic types and STs in populations of the bacterial pathogen *Neisseria meningitidis* [20, 21]. In this paper, we explore whether pneumococcal population structure, and the changes that have occurred since vaccination, can be explained within a similar conceptual framework (Table 1) in which pathogen strains are profiled by antigenic type (AT), metabolic type (MT) and additional non-capsular virulence factors (VF). Strains sharing the same AT experience immunological interference as a consequence of type-specific immunity; interference between strains sharing the same MT or VF may occur through direct competition for similar substrates or for binding to specific host receptors or selected components of the extracellular matrix. Using this framework, we outline the conditions under which associations may arise between antigenic, metabolic and virulence properties of strains within pneumococcal populations and predict how these may shift under vaccination.

**Table 1:** Theoretical framework. Bacterial strains can be conceptualised as aggregates of of antigenic, metabolic and virulence components which are subject to immunological and direct competition.

	Antigenic Type (AT)	Metabolic Type (MT)	Virulence Factors (VF)
**Definition**	Genes encoding targets of protective immunity	Constellation of genes within a bacterium which function in the uptake of nutrients and metabolic processes	Genes associated with increased pathogenicity
**Effect of infection**	Inhibits subsequent infection with strains of the same AT	Inhibits coinfection with strains of the same MP	Inhibits coinfection with strains of the same VT
**Mechanism**	Serotype-specific immunity	Competition for nutrients/receptors (see S1 Supporting Information)	Competition for host binding sites (see S1 Supporting Information)
**Duration of effect**	Lifelong (or shorter if there is waning of immunity)	During carriage	During carriage
**Designation**	a, b, c…	1,2,3…	+/-
**Example**	Capsular serotype	MLST type (see S1 Supporting Information)	Pilus islet

## Results

### Pneumococcal populations contain discrete metabolic types which associate specifically with capsular serotypes

The theoretical framework shown in Table 1 can be applied to a wide range of pathogens, but within the context of *S*. *pneumoniae*, the capsular serotype is the principal determinant of AT. To determine whether pneumococcal populations can also be considered to contain discrete MTs, we interrogated 616 whole pneumococcal genomes published by Croucher *et al*. [22] for allelic differences among 876 metabolic/uptake loci. Associations between alleles of metabolic loci across the genome were found to be highly non-random (Fig 1C), and to exhibit significantly higher levels of linkage disequilibrium (LD) than randomly selected genes not associated with metabolic/transport processes (Fig 1D and 1E). This suggests that a “metabolic profile” may comprise a set of co-evolved genes, which have synergistically adapted to exploit a particular metabolic niche [23], and that such profiles may be stably maintained because any deviation constitutes a loss of fitness [21]. It is possibly that non-metabolic genes contain a higher share of mobile genetic elements but, other than this, we know of no other explanation which could account for the higher levels of association observed between metabolic loci than non-metabolic loci.

We also found the metabolic profiles of isolates belonging to the same ST to be highly concordant (Table F in S1 Text and S1B and S1C Fig). Although a small number of STs shared highly similar metabolic profiles, they also tended to be closely related according to MLST-typing: for example, ST236, ST320 and ST271 manifest highly similar metabolic alleles, and belong to the same MLST-defined clonal complex (CC271). We thus conclude that ST, although defined by only 7 metabolic housekeeping genes, serves as a marker for an extended MT.

We found that metabolic profiles were highly consistent within a serotype (Table D in S1 Text and Fig 1C and S1A Fig), and differed significantly between serotypes (Table E in S1 Text). Certain serotypes were associated with more than one metabolic profile (eg. 6A), and one or two profiles were shared between serotypes (eg. 19A and 15B/C), but the overall pattern was strongly non-overlapping.

### Vaccine induced metabolic shift

**Fig 2:**
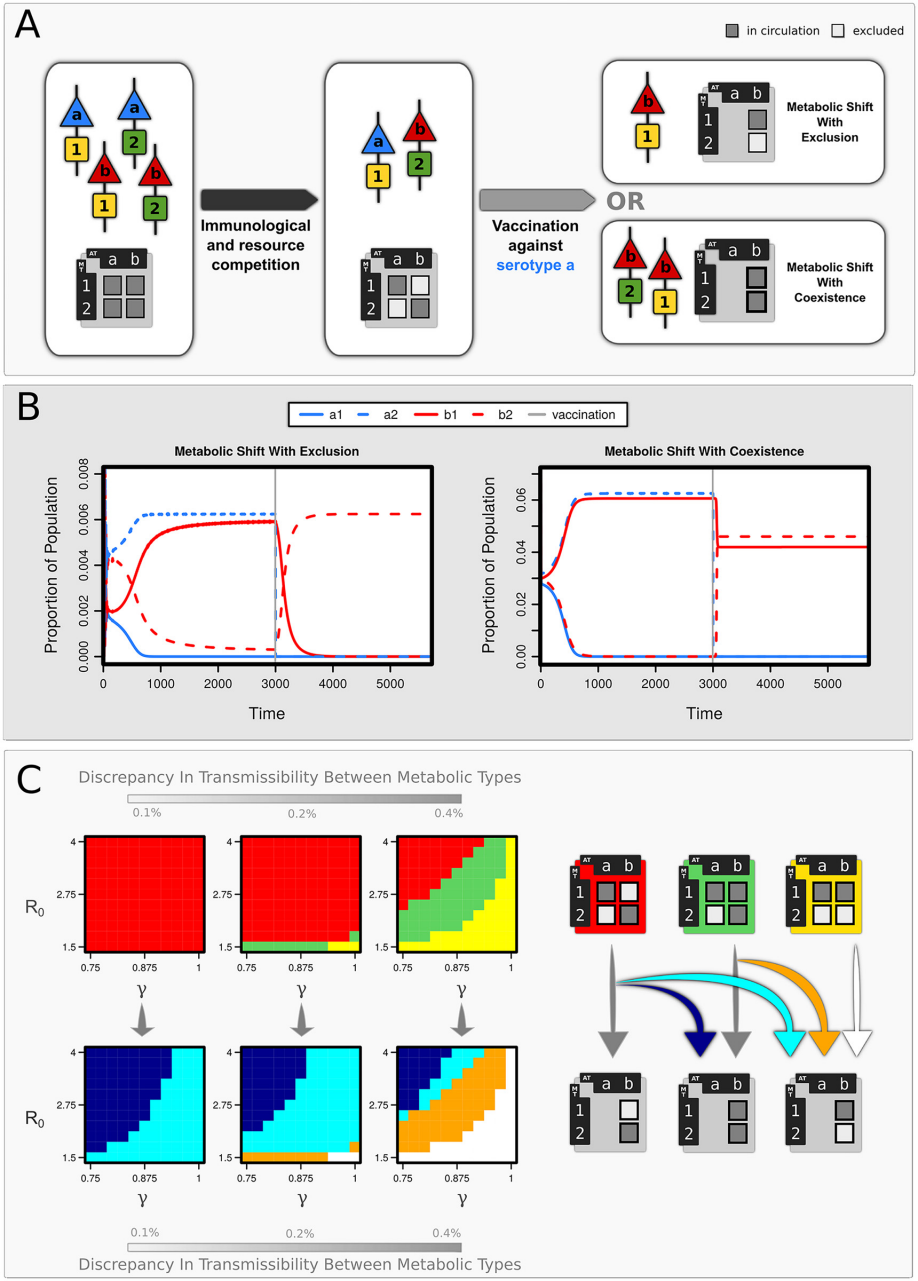
The effects of vaccination on pathogen population structure. (A) Schematic describing how non-overlapping associations emerge between antigenic type (AT: **a,b**) and metabolic type (MT: **1,2**). Vaccination against serotype **a** causes a shift in population structure, inducing a metabolic shift (VIMS) in serotype **b** (B) Strain dynamics following vaccination against serotype **a**, showing two forms of VIMS in serotype **b:** either with exclusion of MT2 (γ = 0.9) or in coexistence with it (γ = 0.75). Other parameters: R_0_ (MT1) = 4.001, R_0_ (MT2) = 4, σ = 4, μ = 0.02. (C) Regions of parameter space associated with (i) non-overlapping associations between AT & MT (red), (ii) competitive exclusion by MT1 (yellow), and (ii) an intermediate state (green). Regions of VIMS are shown in light blue (with exclusion) and dark blue (with coexistence). Orange areas indicate where vaccination has led to the loss of the less transmissible MT (here ***MT2***). Prevalence of a strain had to exceed 5% of the total infections present to be recorded as contributing to the population structure (σ = 4, μ = 0.02).

A range of different population structures can arise within our theoretical framework (Table 1) depending on the strength of serotype-specific immunity (γ) and direct resource competition (ψ) between strains sharing identical metabolic types. Under moderate to high levels of γ and ψ, pneumococcal populations may either (i) be dominated by a highly transmissible MT, (ii) contain a range of similarly transmissible MTs which exhibit non-overlapping associations with existing serotypes, or (iii) exist in a transitional state between these two extremes (Fig 2C, S3, S4 and S5 Figs).

We explored the effects of serotype-specific vaccination within these different scenarios. We observed, under circumstances where serotype and MT exist in non-overlapping combinations (eg. ***a1*** and ***b2***), that vaccination against a particular serotype can lead to the apparent ‘transfer’ of its metabolic types to a non-vaccine serotype (Fig 2A): a phenomenon we term Vaccine-Induced Metabolic Shift (VIMS). This is because vaccination against serotype ***a*** (where ***MT1*** has a slight transmission advantage over ***MT2***) results in the expansion of ***b1*** which, despite possessing a higher transmission efficiency than ***b2***, has been hitherto suppressed by competition with ***a1***. This may or may not lead to the exclusion of ***b2*** depending on the strength of serotype-specific immunity and the difference in transmission efficiency between ***MT1*** and ***MT2***. If both ***b1*** and ***b2*** are present before vaccination (in addition to ***a1***), vaccination against serotype ***a*** tends to lead to the loss of ***MT2*** from the system: in other words, the removal of ***a1*** allows ***b1*** to competitively exclude ***b2***.

These results can be extended to multiple serotypes and MTs (S4 Fig), and also to additional loci that may, for example, encode extra-capsular and extra-metabolic virulence factors (S5 Fig). We find, as above, that the pneumococcal population becomes structured into non-overlapping combinations of serotype, MT and VF as a consequence of interference between strains that share alleles at any of these loci, provided differences in transmissibility between MTs and VFs are not too high and the costs of virulence are low. We observe, for example (S5 Fig), the persistence of only a small subset (***a1+***, ***b2-***, ***c3-***) of the 18 possible variants in a freely recombining pneumococcal population where (***a***, ***b***, ***c***), (***1***, ***2***, ***3***), and (**+,-**) respectively represent variants of AT, MT and VF. This is because, although ***b1+*** may be intrinsically more transmissible than ***b2-***, the latter has the competitive advantage while ***a1+*** is in circulation because it differs from ***a1+*** at all three loci; for similar reasons, ***c3-*** outcompetes ***c1+*, *c1-*, *c2+*, *c2-*** and ***c3+***, even though they may all have higher intrinsic transmissibility. This is consistent with the observation that certain serotypes are over-represented in pneumococcal invasive disease [1,4]. Vaccination against the most ‘virulent’ serotype (here ***a***) promotes the expansion of ***c1+***, which had been hitherto suppressed by competition with ***a1+***. This is because (i) it does not share alleles at the metabolic or virulence loci with the already co-circulating strain **b2-**, and (ii) it outcompetes c3+ (which also does not share alleles with b2-) due to metabolic type 1 having a higher transmission efficiency.

## Discussion

Non-overlapping associations between antigenic and metabolic types are commonly observed in both pneumococcal and other pathogenic bacterial populations (Table B in S1 Text) with, typically, only certain of these combinations being associated with invasive disease [1,4]. Here we demonstrate that this structuring may arise within a theoretical framework containing freely recombining antigenic and metabolic components and virulence factors, due to the combined action of immune selection and direct resource competition.

Does the biology of the pneumococcus fulfil the criteria under which these structures arise in our model? There are few data available on naturally-acquired immunity to pneumococcal carriage, but longitudinal studies [24–26] indicate that serotype-specific immune responses offer protection (though not complete) against further infection. The genomic analysis presented in this paper endorses the view that pneumococcal populations contain discrete metabolic types representing "fitness peaks" of similar heights within a landscape shaped by frequent genetic transfer [21]. Differences in the transporters and uptake systems encoded in the genome dictate specific substrate repertoires for different strains [27,28], therefore strains with identical MTs are likely to experience strong competition within the nutrient-limiting environment of the host nasopharynx. Pneumococcal strains also vary in the relative intensity at which different substrates are fermented [29, 30] and therefore may also compete if they exhibit identical hierarchies of substrate utilisation/fermentation profiles. Our model indicates that a combination of these selective forces can act to structure the pneumococcal populations into non-overlapping combinations of serotype and MT, provided that differences in transmissibility between MTs are small (Fig 2), although wide differences in serotype/MT combinations can exist due to differences in transmission coefficients associated with serotype (S3 Fig). The structuring observed within our framework will not be maintained under high differences among MTs, since the most transmissible type would dominate instead; under these circumstances, the observed associations between serotype and MT would be better explained under processes such as a neutral micro-epidemic evolution [31] or through epistasis [5], although recent analyses [32] indicate the latter is unlikely.

A strength of our framework is that it can also explain why associations between serotype and MT may be intrinsically unstable [33–35] and how vaccination can induce a metabolic shift (VIMS) whereby the MTs of vaccine strains to become associated with non-vaccine serotypes. Our results accord with observations of MLST-defined metabolic types shifting onto non-vaccine strains following the introduction of PCV7 (Fig 1B). Cases of VIMS can also be identified within the whole genome data [22] analysed above. Non-vaccine serotypes 15B/C and 19A can be seen to acquire a new MT sharing at 84.6% (694 out of 820) and 90.0% (737 out of 819) of metabolic loci respectively with vaccine serotype 9V, and an MT with 89.8% (729 out of 812 loci) percentage identity with ST320 in 19F is observed to associate with 19A following vaccination. Positive selection for persistence of metabolic types with new capsules after vaccination also underscores the idea that such metabolic types contain a particularly successful constellation of alleles that allows them to exploit a particular metabolic niche.

It is important to note that these results do not imply that vaccines play a mechanistic role in inducing capsular switching variants; on the contrary, capsular switch variants can be present at low frequencies prior to vaccination but will only expand subsequently due to the removal of competition from vaccine strains. Our model is thus able to resolve why, for example, although the ST695^19A^ vaccine escape variant in the US was first reported in 2003 [11], evolutionary analyses indicate that the capsule switch may have taken place prior to 1997 [22]. Overall, our model predicts that rare genotypes (which may or may not have been there prior to vaccination) may increase in frequency after vaccination due to the removal of ecological competition with vaccine serotypes possessing very similar metabolic profiles.

This transition to a new metabolic profile in non-vaccine serotypes may accompanied by an change in virulence due to a number of reasons: (i) competitive interference from vaccine strains sharing the same virulence factors will have been removed by vaccination (ii) the cost of virulence may be offset by a slight increase in transmissibility due to the acquisition of a different metabolic profile (S4 Fig), and (iii) epistatic interactions between virulence and metabolic types may favour the emergence of virulence on the new metabolic background. A possible example of this phenomenon is the large increase in piliated strains observed in the US since PCV7 vaccination [36, 37]. There are two types of pili in pneumococcus, type I (PI-1) and type II (PI-2), which are found in 30% and 16% of strains respectively [38,39]. In Massachusetts, PI-1 was associated primarily with vaccine-type serotypes before vaccination in 2000. PI-1 subsequently decreased in prevalence with the declining vaccine serotypes, but re-emerged in 2004–2007 in association with non-vaccine serotypes, in particular serotype 19A [36]. Similarly, there has been a 40% increase in PI-2 in serotype 19A following the introduction of PCV7 in Atlanta, Georgia [37]. Significant negative associations have been observed, for example, in co-colonisation of piliated pneumococci and *Staphylococcus aureus* [24]; it would be reasonable to assume that the same type of competition occurs between strains of piliated pneumococci. We also observe, within the WG data, that a number of alleles at loci which have been implicated in increased virulence or pathogenesis were shared between vaccine strains isolated in 2001, and non-vaccine strains isolated after vaccination, in 2004 and 2007 (Table C in S1 Text).

The outgrowth of non-vaccine variants possessing metabolic and virulence factors previously associated with vaccine strains, as predicted by this model, has important implications for the continued success of strain-targeted vaccination programs. Within our framework, drug-resistance alleles may also shift to non-vaccine serotypes due to the removal of competition at these loci, thereby exacerbating the problem. Indeed, in North America, the majority of penicillin-resistant 19A isolates are linked with MLST types (such as ST320) previously associated with vaccine serotypes [6]. Also, in Italy, the highly prevalent antibiotic resistant ST230 clone, previously associated with vaccine serotypes 14 and 19F, is now predominantly observed with non-vaccine serotypes 19A and 24F [40]. As genomic sequencing becomes a routine part of epidemiological surveillance, our theoretical approach—in which strains can be envisaged as comprising a number of interchangeable modular units specifying antigenic, metabolic, virulence and antibiotic resistance properties—can provide a powerful conceptual framework for the analysis of pathogen population biology and of the genomic impact of vaccination programs.

## Materials and Methods

### Metabolic analysis

We explored the allelic variation between metabolic loci in a comprehensive sample of 616 genomes published previously by Croucher *et al*. [22], comprising carriage strains isolated from Massachusetts, USA, in 2001, 2004 and 2007 (see S1 Dataset for the Accession Numbers). Sequence reads were taken from the project ERP000889 on the European Nucleotide Archive (http://www.ebi.ac.uk/) and assembled using an automated pipeline with the Velvet algorithm. Annotation was carried out using the BIGSdb software with an automated BLAST process, and the genomes were analyzed using the Genome Comparator tool (with ATCC 700669 as the reference) [41]. Alleles identical to the Reference Genome were designated as “1”, and subsequent sequences which differ at one or more bases labelled consecutively, and are represented in the S1 Dataset by arbitrary colours with missing alleles shown in black.

We searched through the 2135 identified coding sequences of the reference strain for genes involved in metabolic processes and nutrient uptake. The coding sequences were functionally assigned as “metabolic & transport”, “neither”, or “unknown function” according to the KEGG Orthology (KO) groupings of the KEGG database (Kyoto Encyclopedia of Genes and Genomes; http://www.genome.jp/kegg/). We identified 877 genes involved in metabolic and/or uptake processes.

To explore the structuring of metabolic/uptake alleles among serotypes, we identified the modal allele for each locus and calculated the frequency (or modal percentage identity, MPI) with which it occurred over all the isolates in a given serotype compared to the 10 isolates randomly selected (without replacement) from the total sample (Table D in S1 Text). We also identified a modal metabolic profile (comprising the most frequent alleles found at each locus) for each serotype and the pairwise percentage of alleles which were shared with the modal metabolic profile of other serotypes was calculated (Table E in S1 Text). Similar analyses were performed with respect to ST (S1 Fig).

We used the D’ measure of Linkage Disequilibrium to investigate the strength of associations between pairs of 100 randomly selected metabolic/transport loci across 300 genomes (which were randomly selected for each comparison) compared to 100 randomly selected loci that were not involved in metabolic processes according to the KEGG Orthology categories (www.genome.jp/kegg) (including those, for example, pertaining to genetic information processes such as translation and transcription). D’ was analysed using the 2LD package [42].

### Epidemiological model

We first consider the dynamics of a pathogen population containing two antigenic types (*i* = *a*,*b*) and two metabolic types (*j* = 1,2). We define *y*_*ij*_ to be the proportion of the population infected by strain *ij* (e.g. *a1*), *z*_*i*_ is the proportion of the population immune to serotype *i*, μ is the average host death rate, and σ_*ij*_ is the rate of loss of infectiousness associated with strain *ij*. Let us first assume that infection by a particular strain (e.g. *a1*) cannot occur among individuals who are immune or infected with the same serotype (ie. z_a_). Let us also assume that infection cannot occur among individuals currently infected by other strains with the same metabolic type. Under these circumstances (see S1 Text), the rate of change in the host population infected with strain *a1*, y_*a1*_, and proportion of hosts immune or infected with the same serotype, z_a_, can be given by:

$$\frac{dz_{a}}{dt}=\left(\lambda_{a1}\left(1-y_{b1}\right)+\;\lambda_{a2}\left(1-y_{b2}\right)\right)\left(1-{\text{z}}_{\text{a}}\right)-{\;\mu \text{z}}_{\text{a}}$$ (1)

$$\frac{dy_{a1}}{dt}=\;\lambda_{a1}\left(1-y_{b1}\right)\;\left(1-{\text{z}}_{\text{a}}\right)-{\;\sigma }_{a1}{\text{y}}_{\text{a}1}$$ (2)

Equations for other strains follow a similar form with λ_ij_ = β_ij_y_ij_ and the basic reproduction number R_0_ = β_ij_/σ_ij_ where β_ij_ is the transmission coefficient of strain *ij*. Direct resource competition or strain-specific immunity may be relaxed by modifying the appropriate terms within the equation by a parameter ψ (0 ≤ ψ ≤ **1**) specifying the degree of resistance against co-infection by the same metabolic type and a parameter γ (0 ≤ γ ≤ **1**) specifying the level of strain-specific immunity:

$$\frac{dz_{a}}{dt}=\left(\lambda_{a1}\left(1-\psi y_{b1}\right)+\;\lambda_{a2}\left(1-\psi y_{b2}\right)\right)\left(1-\gamma ({\text{z}}_{\text{a}}-y_{a1}\right)-\;y_{a1})-{\;\mu \text{z}}_{\text{a}}$$ (3)

$$\frac{dy_{a1}}{dt}=\;\lambda_{a1}\left(1-\psi y_{b1}\right)\;\left(1-\gamma ({\text{z}}_{\text{a}}-y_{a1}\right)-\;y_{a1})-{\;\sigma }_{a1}{\text{y}}_{\text{a}1}$$ (4)

When γ = 1, an individual who has previously been infected with strain *a1* cannot subsequently be infected with any strain of antigenic type *a*. When γ = 0, an individual previously infected with strain *a1* remains fully susceptible to all strains of antigenic type *a*. Thus, when γ = 1, we recover eqs (1) and (2) with fully protective type specific immunity while, at γ = 0, the only form of competition in the system for direct resources (ie. through sharing of metabolic type).

This framework may be extended along the same principles to accommodate additional serotypes and metabolic types as well as a third virulence locus, as described in S1 Text.

Vaccination may be included by adding the term v(1- *z*_*j*_) to the rate of change in proportiion immune to *j* if *j* is a serotype [43]. Other methods, such as a stepwise increase in z_j_ may also be employed to the same effect.

## Supporting Information

S1 Fig Analysis of the metabolic/transport alleles among serotypes and MLST-defined STs among 616 pneumococcal isolates sequenced by Croucher *et al*., 2013). (A) Distribution of the modal percentage identity (MPI) of metabolic/uptake alleles for each serotype (the proportion of alleles at each locus which are identical). (B) Partial screenshot of the metabolic profiles of 616 strains, grouped by MLST-defined ST. (C) Distribution of the modal percentage identity (MPI) of metabolic/uptake alleles for the most frequent STs in the dataset.
(TIF)

S2 Fig Distribution of MPI of metabolic/uptake alleles for six serotypes showing relatively diverse metabolic profiles (blue), separated into their respective STs (pink). The MPIs within each STs were much higher than the serotype as a whole, suggesting that some serotypes exhibit more than one metabolic profile STs were assigned to ST groups based on the percentage of identical metabolic/uptake alleles shared at each locus (STs belonging to a given ST group share 80% of their metabolic/uptake alleles). ST groups were named according to the most frequent ST present.
(TIF)

S3 Fig Sensitivity of distribution of model outcomes in Fig 2 (main text) to differences in transmission coefficients associated with capsular serotype or antigenic type (AT). The top panels are identical to Fig 2 in main text (same parameter values); the 2nd and 3rd rows indicate how the distribution of model outcomes changes with increasing difference between intrinsic transmissibility of serotypes (other parameter values remain unchanged).
(TIFF)

S4 Fig Effects of relaxing immunological and direct resource competition. Patterns of association between antigenic *(a*, *b*, *c)* and metabolic *(1*, *2*, *3)* alleles under varying strengths of immunological (γ) and direct resource (ψ) competition. The possible population structures that may arise are shown in pink, orange, blue and grey using the same convention as Fig 2 in main text. No structuring is found within the white areas *{σ* = 3*; β*_*3*_ = 4.5*; ; μ *= 0.02*; β*_*i*_ = *β*_*i-1*_ + *Δβ*, for *i = 1*,*2; Δβ *= 0, 0.0005 and 0.0015in top, middle and lower panels respectively*}*.
(TIF)

S5 Fig Effects of vaccination in a system with additional virulence factors. (A) As a result of immunological and direct resource competition, the population falls into non-overlapping associations between serotype (**a,b,c**), metabolic type (**1,2,3**) and virulence factor (**+,-**). The majority of strains in the original population are competitively excluded, with the surviving strains exhibiting minimal overlap in antigenic, metabolic and virulence alleles (**a1+**, **b2-**, **c3-**). Vaccination against serotype **a** causes a shift in population structure, favoring the increase in frequency of strain **c1+**. (B) Strain dynamics following vaccination against serotype **a**: the previously suppressed strain **c1+** expands and competitively excludes strain **c3-,** thus serotype c increases in both transmission efficiency and virulence potential *{β*_*1*_ =* 4*.*5*, *β*_2_ = 4.9995, *β*_3_ = 4.99, σ_+_ = 3, σ_+_ = 2.997, μ = 0.02*}*. (C) Distribution of population structures (represented using the same convention as Fig 1 with + indicating presence of virulence factor) under small transmission differentials between MT and VF and with increasing cost of virulence *{β*_*1*_ =* 4*.*5*, *β*_*2*_ =* β*_*1*_ *(1+Δβ)*, *β*_*3*_ =* β*_*2*_ *(1+Δβ)* where *Δβ* is the transmission differential between metabolic types; *β*_*_*_ =* 4*.*5*, *β*_+_ =* β*_*_*_ *(1+Δβ*_*v*_ *)* where *Δβ*_*v*_ is the transmission advantage of virulent strains; σ_+_ = 2.99, σ = σ_+_ (1-*Δ*σ) where *Δ*σ is the relative cost of virulence; μ = 0.02}.
(TIFF)

S1 Text Tables A-F and mathematical analyses. (DOCX)

S1 Dataset Metabolic profiles of 616 pneumococcal isolates (obtained from ref 22 in main text). Each column corresponds to a single isolate. Alleles of 876 metabolic/transport genes are represented by arbitrary colours with missing alleles shown in black.
(XLSX)
