# Supplementary figures and images for: Vaccination Drives Changes in Metabolic and Virulence Profiles of Streptococcus pneumoniae
Source: PLoS Pathog. 2015 Jul 16;11(7):e1005034. doi: 10.1371/journal.ppat.1005034 (PMC4504489; doi:10.1371/journal.ppat.1005034)

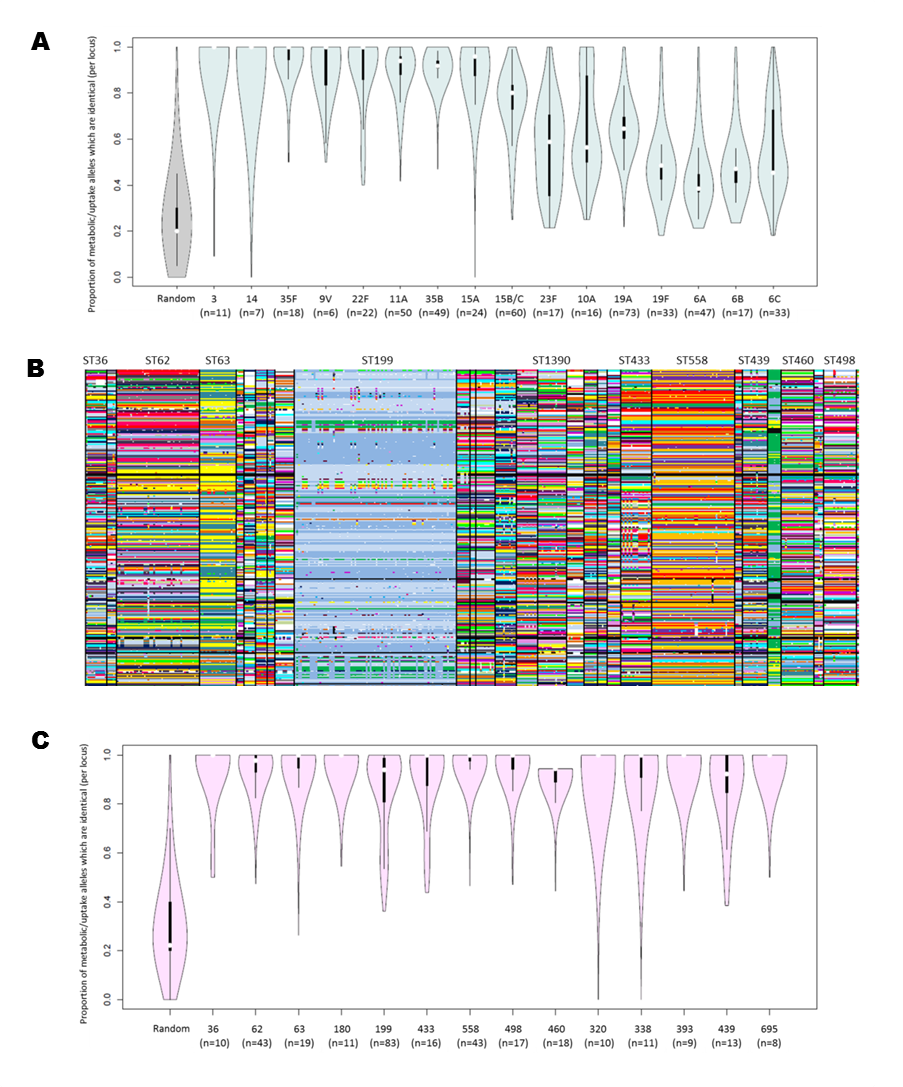

Supplement: S1 Fig — (A) Distribution of the modal percentage identity (MPI) of metabolic/uptake alleles for each serotype (the proportion of alleles at each locus which are identical). (B) Partial screenshot of the metabolic profiles of 616 strains, grouped by MLST-defined ST. (C) Distribution of the modal percentage identity (MPI) of metabolic/uptake alleles for the most frequent STs in the dataset. (TIF) [file ppat.1005034.s001.tif]

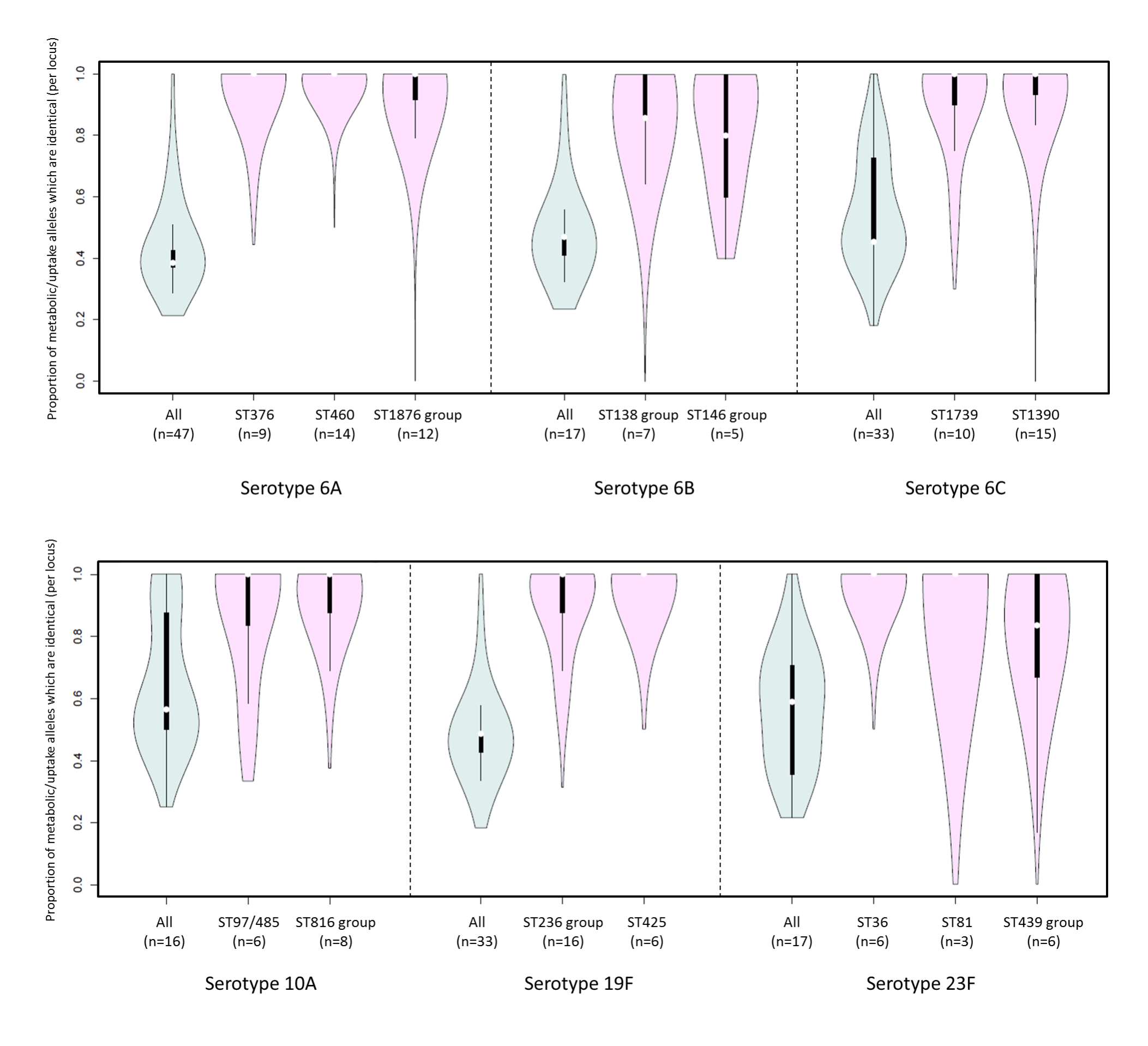

Supplement: S2 Fig — The MPIs within each STs were much higher than the serotype as a whole, suggesting that some serotypes exhibit more than one metabolic profile STs were assigned to ST groups based on the percentage of identical metabolic/uptake alleles shared at each locus (STs belonging to a given ST group share 80% of their metabolic/uptake alleles). ST groups were named according to the most frequent ST present. (TIF) [file ppat.1005034.s002.tif]

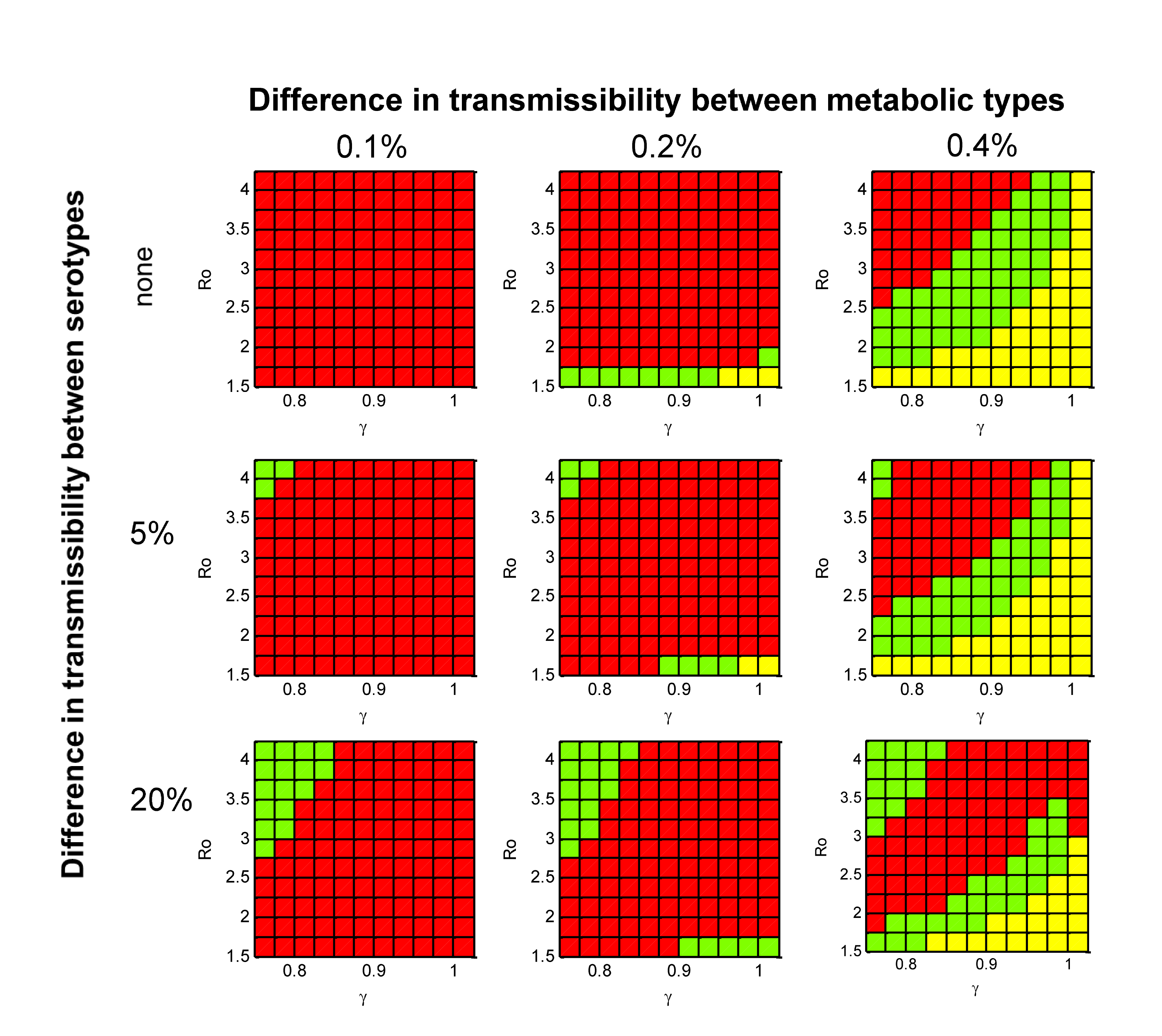

Supplement: S3 Fig — The top panels are identical to Fig 2 in main text (same parameter values); the 2nd and 3rd rows indicate how the distribution of model outcomes changes with increasing difference between intrinsic transmissibility of serotypes (other parameter values remain unchanged). (TIFF) [file ppat.1005034.s003.tiff]

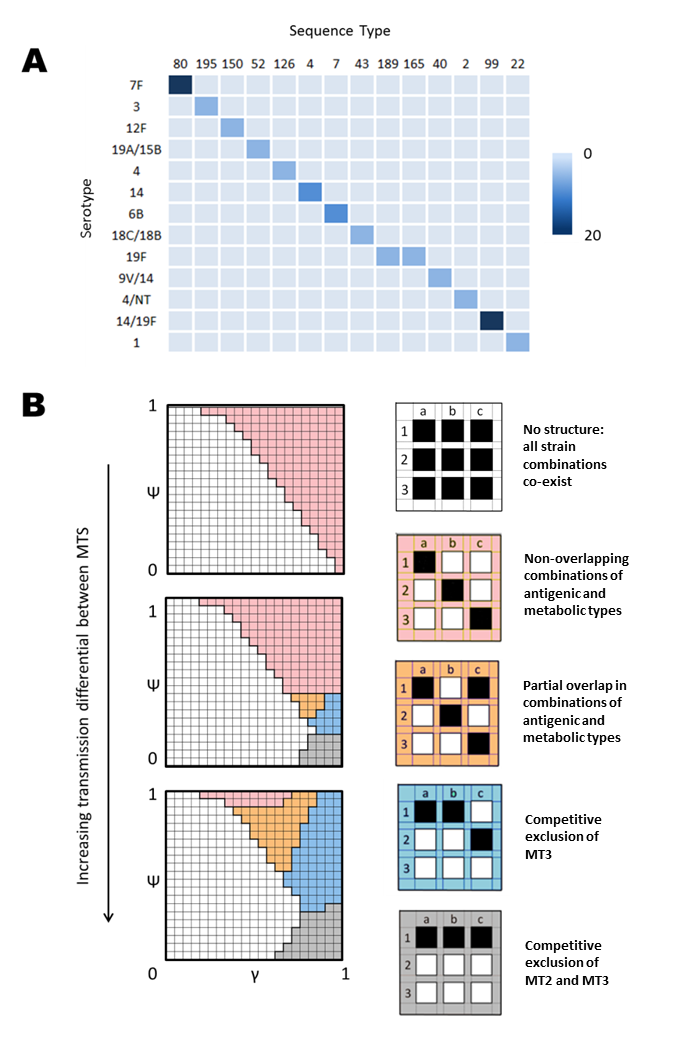

Supplement: S4 Fig — Patterns of association between antigenic (a, b, c) and metabolic (1, 2, 3) alleles under varying strengths of immunological (γ) and direct resource (ψ) competition. The possible population structures that may arise are shown in pink, orange, blue and grey using the same convention as Fig 2 in main text. No structuring is found within the white areas {σ = 3; β 3 = 4.5; ; μ = 0.02; β i = β i-1 + Δβ, for i = 1,2; Δβ = 0, 0.0005 and 0.0015in top, middle and lower panels respectively}. (TIF) [file ppat.1005034.s004.tif]

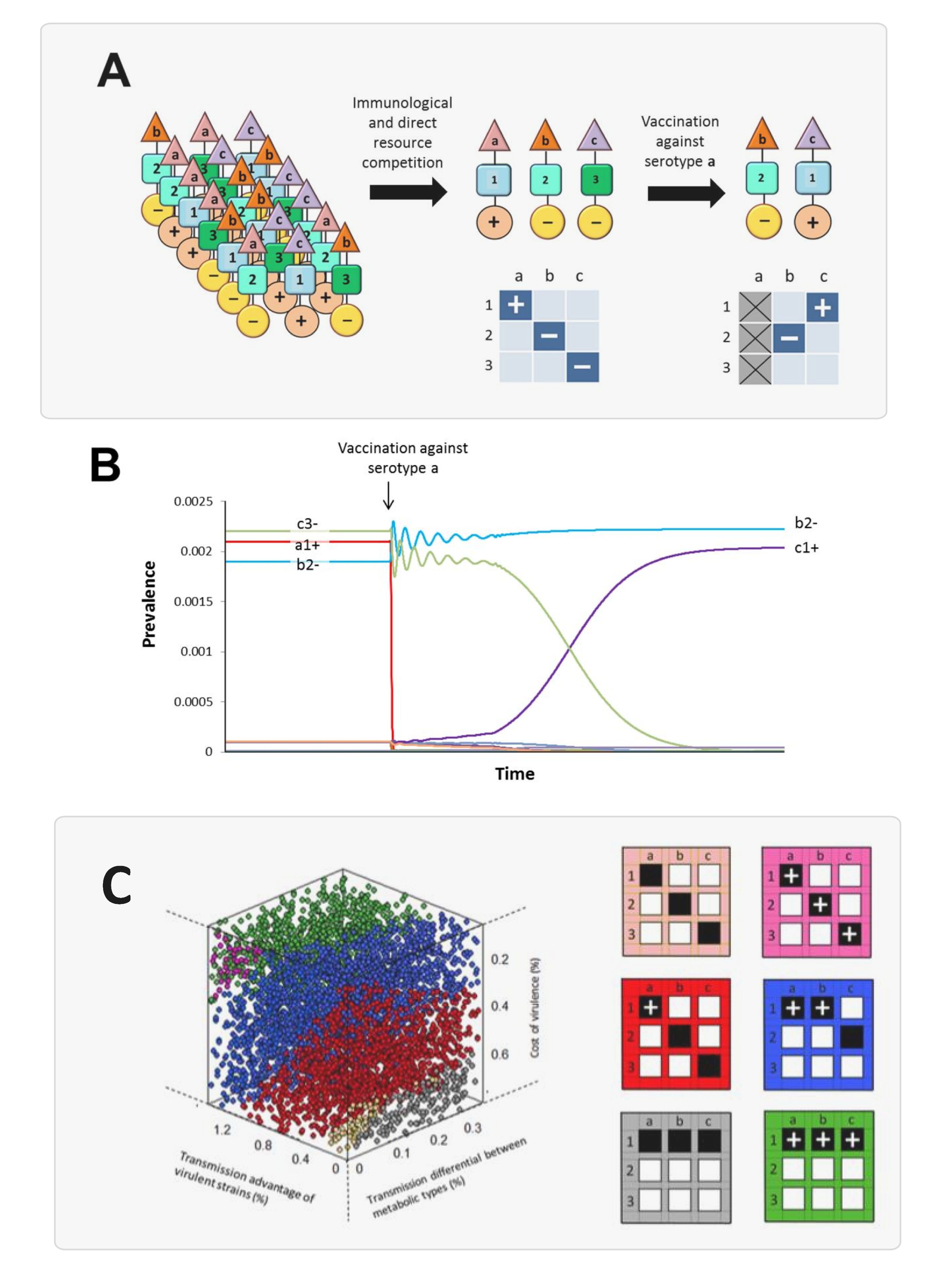

Supplement: S5 Fig — (A) As a result of immunological and direct resource competition, the population falls into non-overlapping associations between serotype (a,b,c), metabolic type (1,2,3) and virulence factor (+,-). The majority of strains in the original population are competitively excluded, with the surviving strains exhibiting minimal overlap in antigenic, metabolic and virulence alleles (a1+, b2-, c3-). Vaccination against serotype a causes a shift in population structure, favoring the increase in frequency of strain c1+. (B) Strain dynamics following vaccination against serotype a: the previously suppressed strain c1+ expands and competitively excludes strain c3-, thus serotype c increases in both transmission efficiency and virulence potential {β 1 = 4.5, β 2 = 4.9995, β 3 = 4.99, σ+ = 3, σ+ = 2.997, μ = 0.02}. (C) Distribution of population structures (represented using the same convention as Fig 1 with + indicating presence of virulence factor) under small transmission differentials between MT and VF and with increasing cost of virulence {β 1 = 4.5, β 2 = β 1 (1+Δβ), β 3 = β 2 (1+Δβ) where Δβ is the transmission differential between metabolic types; β _ = 4.5, β + = β _ (1+Δβ v ) where Δβ v is the transmission advantage of virulent strains; σ+ = 2.99, σ = σ+ (1-Δσ) where Δσ is the relative cost of virulence; μ = 0.02}. (TIFF) [file ppat.1005034.s005.tiff]
