# Supplementary material for: Vaccination Drives Changes in Metabolic and Virulence Profiles of Streptococcus pneumoniae
Source: PLoS Pathog. 2015 Jul 16;11(7):e1005034. doi: 10.1371/journal.ppat.1005034 (PMC4504489; doi:10.1371/journal.ppat.1005034)
Supplement: S1 Text — (DOCX) [file ppat.1005034.s006.docx]

**SUPPLEMENTARY TABLES**

**Table** **A:** Studies in which non-overlapping associations between serotype and markers of metabolic type (ST = Multi Locus Sequence Type (1); ET = Multi Locus Enzyme Electrophoresis type (2)) were observed among *Streptococcus pneumoniae.*

| Metabolic type | Country/countries included in study | No. isolates | Reference |
| --- | --- | --- | --- |
|  |  |  |  |
| ST | USA | 616 | (3) |
| ST | Scotland | 217 | (4) |
| ST | Australia, Canada, Denmark, Finland, the Netherlands, Sweden, Great Britain and Uruguay | 274 | (5) |
|  |  |  |  |
| ST | UK | 30 | (6) |
|  |  |  |  |
| ST | UK | 501 | (7) |
|  |  |  |  |
| ST | UK | 310 | (8) |
| ST | USA | 1168 | (9) |
|  |  |  |  |
| ST | Finland | 224 | (10) |
| ST | Finland | 437 | (11) |
|  |  |  |  |
| ST | Columbia | 629 | (12) |
| ST  ST  ST  ET | UK  Japan  Taiwan  Spain, Hungary, USA | 1030  66  68  342 | (13)  (14)  (15)  (49) |
|  |  |  |  |

**Table** **B:** Associations among antigenic and metabolic genes for 17 pathogenic and non-pathogenic bacterial species. ST refers to sequence type; ET refers to electrophoretic type. Clonal complex refers to a group of highly similar STs which share identical MLST alleles at several loci.

| Pathogen | Antigenic Type | Metabolic Type | No. isolates | Reference |
| --- | --- | --- | --- | --- |
| *Neisseria meningitidis* | PorA (outer membrane protein) | Clonal complex | 977 | (17) |
|  | PorA, FetA (outer membrane proteins) | Clonal complex | 3460 | (18) |
|  | fHbp (factor H binding protein) | Clonal complex | 107 | (19) |
|  | Opa (opacity related protein) | Clonal complex | 77 | (20) |
| *Staphylococcus aureus* | Spa types (Staphylococcal Protein A), ClfA & ClfB (clumping factor A & B) | Clonal complex | 224 | (21) |
|  | Spa types | ST | 182 | (22) |
| *Streptococcus pyogenes* | Emm types (M protein) | ST | 495 | (23) |
|  | Emm types (M protein) | ST | 212 | (24) |
| *Salmonella enterica* | Serovar (O and H antigens) | eBGs (eBurstGroups) | 4257 | (25) |
| *Haemophilus*  *influenzae* | Serotype | ET | 2209 | (26) |
|  | Serotype | Clonal complex | 131 | (27) |
| *Helicobacter pylori* | CagA | ST | 129 | (28) |
| *Eschericha coli* | O antigen (lipopolysaccharide) | ET | 187 | (29) |
|  |  |  |  |  |
| *Clostridium difficile* | Surface-Associated Protein A (slpA) | ST | 42 | (30) |
| *Listeria monocytogenes* | Serotype (somatic (O) antigen and flagellar (H) antigen) | ET | 175 | (31) |
| *Neisseria gonorrhoeae* | 11 Opa genes (opacity related protein) | ST | 14 | (32) |
|  |  |  |  |  |
| *Klebsiella pneumoniae* | Serotype (C pattern) | ST | 63 | (33) |
| *Campylobacter jejuni* | porA (outer membrane protein) | Clonal complex | 584 | (34) |
| *Neisseria lactamica* | FetA | ST | 275 | (35) |
|  |  |  |  |  |
| *Enterococcus faecium* | ace, salA, lsa (collagen/laminin adhesin; a cell wall-associated antigen; putative ABC transporter) | ST | 50 | (36) |
| *Streptococcus dysgalactiae subsp. Equisimilis* | Emm types (M protein) | ST | 334 | (37) |
| *Pasteurella trehalosi* | Serotype, outer membrane protein, LPS | ET | 60 | (38) |
| *Capnocytophaga species* | igA protease | ET | 50 | (39) |

**Table** **C:** Alleles of 21 virulence-associated loci that have increased in frequency (%) among non-vaccine strains, potentially through VIMS. T refers to alleles which have been truncated; X refers to alleles not present in the genomic assembly.

|  |  | Vaccine strain | Non-vaccine strains | | Vaccine strain | Non-vaccine strains | | |
| --- | --- | --- | --- | --- | --- | --- | --- | --- |
| Locus | Genome position | 19F ST320 | 19A ST320 | 19A ST199 | 9V ST156 | 19A ST1925 | 15/AB ST162 or 3275 | 15A/B ST199 |
| nanA \| SPN23F12210 | 1189320 | 29 (0.9) | 29 (100) | 3 (0.91) | 8 (100) | 8 (100) | 2 (100) | 2 (0.81) |
| bgaA \| SPN23F05830 | 571752 | 21 (100) | 21 (100) | 2 (0.91) | 9 (100) | 9 (100) | 9 (100) | 2 (0.65) |
| strH \| SPN23F00730 | 69102 | 45 (0.9) | 45 (100) | 1 (0.8) | 23 (0.6) | 23 (100) | 23 (0.75) | 1 (0.83) |
| SPN23F02890 | 274732 | 19 (100) | 81 (100) | 1 (0.77) | 1 (0.8) | 1 (100) | 42 (100) | 2 (0.6) |
| fbpS \| pavA \| SPN23F08910 | 864927 | 17 (100) | 17 (100) | 2 (100) | 4 (100) | 4 (100) | 4 (100) | 2 (0.81) |
| eno \| SPN23F10490 | 1012087 | 13 (100) | 13 (100) | 1 (100) | 8 (100) | 8 (100) | 8 (100) | 1 (100) |
| ply \| SPN23F19470 | 1889503 | 12 (100) | 12 (100) | 2 (100) | 8 (0.8) | 8 (100) | 8 (100) | 2 (0.96) |
| lytA \| SPN23F19600 | 1898038 | 1 (100) | 1 (100) | 3 (0.86) | 9 (100) | 3 (100) | 9 (50) | 3 (0.75) |
| nanB \| SPN23F16870 | 1626581 | 37 (100) | 37 (100) | 3 (0.91) | 10 (100) | 10 (100) | 10 (100) | 3 (0.65) |
| SPN23F06940 | 681761 | 12 (100) | 12 (100) | 1 (100) | 8 (100) | 8 (100) | 8 (100) | 1 (0.92) |
| lytB \| SPN23F08900 | 862890 | 59 (100) | 59 (100) | 1 (100) | 11 (100) | 11 (100) | 11 (100) | 1 (0.63) |
| cbpE \| SPN23F08530 | 834686 | 30 (100) | 30 (100) | 1 (0.94) | 9 (100) | 9 (100) | 9 (100) | 1 (100) |
| phtE \| SPN23F09300 | 898830 | 52 (100) | 23 (100) | 2 (0.94) | 3 (100) | 3 (100) | 3 (100) | 2 (0.94) |
| gapN \| SPN23F10400 | 1002438 | 17 (100) | 17 (100) | 1 (0.94) | 9 (100) | 9 (100) | 9 (75) | 1 (0.98) |
| SPN23F04520 | 435720 | 23 (100) | 23 (100) | 26 (0.91) | 12 (100) | 47 (100) | 52 (100) | 2 (0.6) |
| ccpA \| SPN23F20200 | 1959921 | 4 (100) | 4 (100) | 1 (100) | 1 (100) | 1 (100) | 1 (100) | 1 (0.98) |
| pcpA \| SPN23F21690 | 2108466 | 23 (0.22) | 15 (50) | 28 (0.14) | T (0.8) | T (50) | 62 (50) | 3 (0.1) |
| cbpD \| SPN23F22340 | 2181850 | X (100) | 29 (100) | 1 (0.97) | 8 (0.8) | 8 (0.75) | 8 (100) | 1 (0.71) |
| htrA \| SPN23F22720 | 2219291 | 4 (100) | 4 (100) | 2 (100) | 8 (100) | 8 (100) | 8 (75) | 2 (100) |
| cbpJ \| SPN23F03490 | 340640 | 33 (0.67) | 33(100) | 7 (0.91) | 1 (100) | 7 (100) | 20 (100) | 2 (0.21) |
| cbpG \| SPN23F03640 | 352443 | 39 (0.67) | 39(100) | 21 (0.97) | 10 (100) | 21 (100) | 10 (100) | 2 (0.94) |

**Table D:** Outputs of the two-tailed Sign Tests comparing the average proportion of metabolic/transport alleles which are identical at a given locus (MPI), between isolates of the same serotype and randomly-selected isolates. The tests were performed for the most common serotypes in the dataset published by Croucher *et al.*(1). The sample estimate refers to the point estimate of the median difference between the two lists of modal percentage identities.

| Test | No. positive differences | No. negative differences | Ties | Test statistic | Standardised test statistic | Sample estimate | Upper achieved confidence interval (0.9509) | P-value (2-sided) |
| --- | --- | --- | --- | --- | --- | --- | --- | --- |
| 3 - Random | 770 | 10 | 2 | 770 | 27.17 | 0.71 | 0.71 - 0.80 | < 2.2e-16 |
| 14 - Random | 777 | 3 | 2 | 777 | 27.68 | 0.70 | 0.70 - 0.78 | < 2.2e-16 |
| 35F - Random | 780 | 0 | 2 | 780 | 27.89 | 0.74 | 0.70 - 0.74 | < 2.2e-16 |
| 9V - Random | 764 | 15 | 3 | 764 | 26.8 | 0.70 | 0.70 - 0.70 | < 2.2e-16 |
| 22F - Random | 778 | 2 | 2 | 778 | 27.75 | 0.70 | 0.70 - 0.70 | < 2.2e-16 |
| 11A - Random | 778 | 1 | 3 | 778 | 27.8 | 0.66 | 0.66 - 0.68 | < 2.2e-16 |
| 35B - Random | 780 | 0 | 2 | 780 | 27.89 | 0.70 | 0.69 - 0.70 | < 2.2e-16 |
| 15A - Random | 775 | 2 | 5 | 775 | 27.7 | 0.66 | 0.66 - 0.70 | < 2.2e-16 |
| 15B/C - Random | 769 | 10 | 3 | 769 | 27.16 | 0.53 | 0.52 - 0.53 | < 2.2e-16 |
| 23F - Random | 756 | 18 | 8 | 756 | 26.49 | 0.38 | 0.35 - 0.40 | < 2.2e-16 |
| 19A - Random | 770 | 11 | 1 | 770 | 27.12 | 0.41 | 0.40 - 0.42 | < 2.2e-16 |
| 19F - Random | 772 | 58 | 2 | 772 | 23.74 | 0.25 | 0.23 - 0.25 | < 2.2e-16 |
| 10A - Random | 748 | 20 | 14 | 748 | 26.23 | 0.36 | 0.31 - 0.36 | < 2.2e-16 |
| 6B - Random | 726 | 55 | 1 | 726 | 23.97 | 0.22 | 0.22 - 0.22 | < 2.2e-16 |
| 6C - Random | 741 | 39 | 2 | 741 | 25.1 | 0.25 | 0.25 - 0.25 | < 2.2e-16 |
| 6A - Random | 687 | 93 | 2 | 687 | 21.23 | 0.18 | 0.18 - 0.18 | < 2.2e-16 |

**Table E:** The percentage of metabolic/uptake alleles shared between pairs of serotypes. Loci in which alleles were truncated or absent were excluded from the analysis, providing a total of 756 loci. Serotypes comprising more than one metabolic type were excluded, as the modal metabolic profile is not a legitimate representation of the metabolic traits for these serotypes. The percentage of identical metabolic/uptake alleles shared across serotypes was relatively low (with any two serotypes sharing 10.1% of their metabolic/uptake alleles on average), and significantly less than 1 (Wilcoxon Mann Whitney test, V = 1540, p-value < 1.129e-10).

|  | **3** | **14** | **11A** | **15A** | **15B/C** | **19A** | **22F** | **23F** | **35F** | **9V** | **35B** |
| --- | --- | --- | --- | --- | --- | --- | --- | --- | --- | --- | --- |
| **3** | 1 | 0.077 | 0.085 | 0.057 | 0.070 | 0.077 | 0.070 | 0.085 | 0.089 | 0.028 | 0.078 |
| **14** | 0.077 | 1 | 0.120 | 0.104 | 0.097 | 0.131 | 0.083 | 0.086 | 0.079 | 0.074 | 0.093 |
| **11A** | 0.085 | 0.120 | 1 | 0.192 | 0.112 | 0.110 | 0.091 | 0.083 | 0.122 | 0.078 | 0.097 |
| **15A** | 0.057 | 0.104 | 0.192 | 1 | 0.091 | 0.102 | 0.079 | 0.085 | 0.094 | 0.077 | 0.093 |
| **15B/C** | 0.070 | 0.097 | 0.112 | 0.091 | 1 | 0.806 | 0.106 | 0.142 | 0.086 | 0.103 | 0.104 |
| **19A** | 0.077 | 0.131 | 0.110 | 0.102 | 0.806 | 1 | 0.107 | 0.143 | 0.077 | 0.120 | 0.106 |
| **22F** | 0.070 | 0.083 | 0.091 | 0.079 | 0.106 | 0.107 | 1 | 0.110 | 0.094 | 0.075 | 0.146 |
| **23F** | 0.085 | 0.086 | 0.083 | 0.085 | 0.142 | 0.143 | 0.110 | 1 | 0.085 | 0.090 | 0.091 |
| **35F** | 0.089 | 0.079 | 0.122 | 0.094 | 0.086 | 0.077 | 0.094 | 0.085 | 1 | 0.086 | 0.097 |
| **9V** | 0.074 | 0.074 | 0.078 | 0.077 | 0.103 | 0.120 | 0.075 | 0.090 | 0.086 | 1 | 0.078 |
| **35B** | 0.078 | 0.093 | 0.097 | 0.093 | 0.104 | 0.106 | 0.146 | 0.091 | 0.097 | 0.078 | 1 |

**Table F:** Outputs of the two-tailed Sign Tests comparing the average proportion of metabolic/transport alleles which are identical at a given locus (MPI), between isolates of the same ST and randomly-selected isolates. The tests were performed for the most common STs in the dataset published by Croucher *et al.*(3). The sample estimate refers to the point estimate of the median difference between the two lists of modal percentage identities.

| Test | No. positive differences | No. negative differences | Ties | Test statistic | Standardised test statistic | Sample estimate | Upper achieved confidence interval (0.9566) | P-value (2-sided) |
| --- | --- | --- | --- | --- | --- | --- | --- | --- |
| ST36 - Random | 758 | 2 | 9 | 758 | 27.39 | 0.70 | 0.70 - 0.70 | < 2.2e-16 |
| ST62 - Random | 762 | 3 | 4 | 762 | 27.41 | 0.68 | 0.66 - 0.68 | < 2.2e-16 |
| ST63 - Random | 762 | 2 | 5 | 762 | 27.46 | 0.70 | 0.70 - 0.70 | < 2.2e-16 |
| ST180- Random | 763 | 0 | 6 | 763 | 27.59 | 0.70 | 0.70 - 0.70 | < 2.2e-16 |
| ST199 - Random | 754 | 13 | 2 | 754 | 26.72 | 0.62 | 0.60 - 0.64 | < 2.2e-16 |
| ST433 - Random | 757 | 6 | 6 | 757 | 27.15 | 0.68 | 0.64 - 0.70 | < 2.2e-16 |
| ST558 - Random | 763 | 2 | 4 | 763 | 27.48 | 0.70 | 0.70 - 0.71 | < 2.2e-16 |
| ST498 - Random | 763 | 0 | 6 | 763 | 27.58 | 0.70 | 0.70 - 0.70 | < 2.2e-16 |
| ST460 - Random | 763 | 6 | 0 | 763 | 27.26 | 0.64 | 0.64 - 0.64 | < 2.2e-16 |
| ST320 - Random | 760 | 3 | 6 | 760 | 27.37 | 0.70 | 0.70 - 0.70 | < 2.2e-16 |
| ST338 - Random | 758 | 6 | 5 | 758 | 27.17 | 0.62 | 0.62 - 0.70 | < 2.2e-16 |
| ST393 - Random | 763 | 1 | 5 | 763 | 27.53 | 0.70 | 0.70 - 0.70 | < 2.2e-16 |
| ST439 - Random | 760 | 5 | 5 | 760 | 27.26 | 0.62 | 0.60 - 0.65 | < 2.2e-16 |
| ST695 - Random | 763 | 0 | 6 | 763 | 27.59 | 0.70 | 0.70 - 0.78 | < 2.2e-16 |

**Epidemiological Model**

The following diagram shows an SIR based representation of a system with two antigenic alleles (e.g. capsular serotypes), *a* and *b*, and two metabolic alleles, 1 and 2, thus yielding a total of four possible strains (*a1, a2, b1* and *b2*). The host population is divided into a susceptible class (S), ten infected classes (Y_a1_, Y_a2_, Y_b1_, Y_b2_, Y_a1b2_, Y_a2b1_; V_a1_, V_a2_, V_b1_, V_b2_), and three immune classes (Z_a_, Z_b_; Z_ab_). For clarity, the host birth and death processes are not shown. The shaded area contains all individuals who have been infected with and are subsequently no longer susceptible to serotype *a*.

Susceptible hosts (S) become infected with strain *a1*, for example, at the rate λ_a1_= β_a1_ (Y_a1_+ Y_a1b2_ +V_a1_) where Y and V refer to primary and secondary infections with designated strains/strain combinations and β_a1_ is a transmission coefficient. Recovery occurs at a rate σ and leads to serotype-specific immunity, such that individuals in Z_a_ can no longer be infected by a strain of serotype *a*, and individuals in Z_ab_ are immune to both *a* and *b*.

Additionally, individuals who are currently infected by a particular metabolic type cannot be co-infected by a strain with the same metabolic type due to direct resource competition. Thus it is possible to move from Y_a1_ to Y_a1b2_ but not to Y_a1b1_. As an example, a susceptible individual (S) who becomes infected with strain *a1* (Y_a1_) can either: (i) become co-infected with strain *b2* (Y_a1b2_) and move directly to Z_ab_, or (ii) clear the infection and gain immunity to a (Z_a_), before later becoming infected with *b1* (V_b1_) or *b2* (V_b2_). Through either route, the individual can ultimately gain immunity to both *a* and *b* (Z_ab_).

SIR formulations do not lend themselves to extension to multiple strains and many different srategies have been developed to overcome this problem (40). Here, we use an overlapping compartmental framework developed by Gupta et al (41) to recast these equations in the following manner:

Let *z_a_* contain all individuals who have been infected with antigenic type *a*, as shown by the shaded area in the flow diagram, and *y_a1_* contain all individuals currently infected with *a1*:

$${\frac{{dz}_{a}}{dt}=\lambda_{a1}\left( 1-z_{a}- Y_{b1} \right)+\lambda_{a2}\left( 1-z_{a}- Y_{b2} \right)- \mu z}_{a} (S1)$$

$${\frac{{dy}_{a1}}{dt}= \lambda_{a1}\left( 1-z_{a}-Y_{b1} \right)- \sigma y}_{a1} (S2)$$

Since *y_a1_= Y_a1_ + Y_a1b2_ + V_a1_,* we obtain:

$${\frac{{dy}_{a1}}{dt}= \lambda_{a1}\left( S + Y_{b2} + Z_{b} \right)- \sigma y}_{a1}$$

by adding:

$${\frac{{dY}_{a1}}{dt}= \lambda_{a1}S-(\sigma+\lambda_{b2})Y}_{a1}$$

$${\frac{{dY}_{a1b2}}{dt}= \lambda_{a1}Y_{b2}{+\lambda}_{b2}Y_{a1}-\sigma Y}_{a1b2}$$

$${\frac{{dV}_{a1}}{dt}= \lambda_{a1}Z_{b}-\sigma V}_{a1}$$

Since S + Y*_b2_* + Z**_b_** = 1- *z_a_* - Y_b1_ = 1-*z_a_* - *y_b1_*(1-*z_a_* - *y_a1_*) we can write:

| ${\frac{{dz}_{a}}{dt}=\sum_{j=1,2} \lambda_{aj}[\left( 1-y_{bj} \right) \left( 1-z_{a} \right)-y_{aj}y_{bj}] - \mu z}_{a}$ | (S3) |
| --- | --- |
| ${\frac{{dy}_{a1}}{dt}= \lambda_{a1}[\left( 1-y_{b1} \right) \left( 1-z_{a} \right)-y_{a1}y_{b1}]- \sigma_{a1}y}_{a1}$ | (S4) |

Equations for other strains follow a similar form with λ_ij_= β_ij_y_ij_ and the basic reproduction number R_0_ = β_ij_/σ_ij_ with β_ij_= *f*(β_i,_ β_j_) and σ_ij_ = *g*(σ_i_, σ_j_) where *i* indicates it is a property associated with AT, and *j* with MT. These may be simplified further under the approximation: *y_b1_*(1-*z_a_* - *y_a1_*) ≈ *y_b1_*(1-*z_a_*), yielding eqns (1-2) in the main text .

This model may be extended to relax the strength of immunological and direct resource competition by introducing the parameters ψ and γ, respectively specifying the degree of resistance against co-infection by the same metabolic type and the level of strain-specific immunity.

| ${\frac{{dz}_{a}}{dt}= \sum_{j=1,2} \lambda_{aj} \left( 1-{\gamma(z}_{a}-y_{aj} \right)- y_{aj} -\psi y_{bj}(\left( 1-z_{a} \right) - y_{aj})]]- \mu z}_{a}$ | (S5) |
| --- | --- |
| ${\frac{{dy}_{a1}}{dt}= \lambda_{a1} \left( 1-{\gamma(z}_{a}-y_{a1} \right)- y_{a1} -\psi y_{b1}(\left( 1-z_{a} \right) - y_{a1})]- \sigma_{a1}y}_{a1}$ | (S6) |

Once again these may be further simplified using the approximation: *y_b1_*(1-*z_a_* - *y_a1_*) ≈ *y_b1_*(1-*z_a_*).

For the case ψ = γ = 1, we were able to validate the results using the SIR framework, and these were also in agreement with those obtained from a stochastic model containing the same assumptions published by Buckee *et al*. (17). In the latter, the antigenic type was characterised by multiple loci, which allowed transitions between different non-overlapping states to occur. This dynamical behavior may explain some of the natural historical variation in pneumococcal populations (41), but for the purposes of elucidating the effects of vaccination we stick to a single antigenic locus in our analysis.

This framework may be extended to accommodate additional alleles or to accomodate non-capsular virulence factors in the following manner:

| $\frac{{dy}_{i}}{dt}= \lambda_{i} \left( 1-z_{j} \right)\prod_{k} (1-y_{k})- \sigma_{i}y_{i}$ | (S7) |
| --- | --- |
|  |  |
| ${\frac{{dz}_{j}}{dt}=\left( 1-z_{j} \right)\sum_{i} \lambda_{i}\prod_{k} (1-y_{k})- \mu z}_{j}$ | (S8) |
|  |  |

We assume that infection by a particular strain (e.g. *a1+*) can only occur among individuals who are not immune or infected with the same serotype (i.e. 1-z_a_), thus *j* represents the serotype of strain *i*. We also assume that infection cannot occur among individuals currently infected by other strains with either the same metabolic type or virulence factor (denoted in the equations above by *k*) and encompassing, for example *b1+, b2+, b1-, c1+, c1-, etc* for strain *a1+*.

**Supporting References**

1. Maiden MCJ, Bygraves JA, Feil E, Morelli G, Russell JE, Urwin R, et al. Multilocus sequence typing: A portable approach to the identification of clones within populations of pathogenic microorganisms. Proceedings of the National Academy of Sciences of the United States of America. 1998;95(6):3140-5. doi: 10.1073/pnas.95.6.3140. PubMed PMID: WOS:000072596200078.

2. Selander RK, Caugant DA, Ochman H, Musser JM, Gilmour MN, Whittam TS. METHODS OF MULTILOCUS ENZYME ELECTROPHORESIS FOR BACTERIAL POPULATION-GENETICS AND SYSTEMATICS. Applied and Environmental Microbiology. 1986;51(5):873-84. PubMed PMID: WOS:A1986C240800001.

3. Croucher NJ, Finkelstein JA, Pelton SI, Mitchell PK, Lee GM, Parkhill J, et al. Population genomics of post-vaccine changes in pneumococcal epidemiology. Nature Genetics. 2013;45(6):656-+. doi: 10.1038/ng.2625. PubMed PMID: WOS:000319563900013.

4. Cobey S, Lipsitch M. Niche and Neutral Effects of Acquired Immunity Permit Coexistence of Pneumococcal Serotypes. Science. 2012;335(6074):1376-80. doi: 10.1126/science.1215947. PubMed PMID: WOS:000301531600055.

5. Enright MC, Spratt BG. A multilocus sequence typing scheme for Streptococcus pneumoniae: identification of clones associated with serious invasive disease. Microbiology-Uk. 1998;144:3049-60. PubMed PMID: WOS:000076975400013.

6. Enright MC, Knox K, Griffiths D, Crook DW, Spratt BG. Molecular typing of bacteria directly from cerebrospinal fluid. Eur J Clin Microbiol Infect Dis. 2000;19(8):627-30. PubMed PMID: 11014627.

7. Brueggemann AB, Griffiths DT, Meats E, Peto T, Crook DW, Spratt BG. Clonal relationships between invasive and carriage Streptococcus pneumoniae and serotype- and clone-specific differences in invasive disease potential. Journal of Infectious Diseases. 2003;187(9):1424-32. doi: 10.1086/374624. PubMed PMID: WOS:000182273700010.

8. Tocheva AS, Jefferies JMC, Christodoulides M, Faust SN, Clarke SC. Distribution of carried pneumococcal clones in UK children following the introduction of the 7-valent pneumococcal conjugate vaccine: A 3-year cross-sectional population based analysis. Vaccine. 2013;31(31):3187-90. doi: 10.1016/j.vaccine.2013.04.075. PubMed PMID: WOS:000321417600013.

9. Beall B, McEllistrem MC, Gertz RE, Wedel S, Boxrud DJ, Gonzalez AL, et al. Pre- and postvaccination clonal compositions of invasive pneumococcal serotypes for isolates collected in the United States in 1999, 2001, and 2002. Journal of Clinical Microbiology. 2006;44(3):999-1017. doi: 10.1128/jcm.44.3.999-1017.2006. PubMed PMID: WOS:000236095000051.

10. Hanage WP, Kaijalainen TH, Syrjanen RK, Auranen K, Leinonen M, Makela PH, et al. Invasiveness of serotypes and clones of Streptococcus pneumoniae among children in Finland. Infection and Immunity. 2005;73(1):431-5. doi: 10.1128/iai.73.1.431-435.2005. PubMed PMID: WOS:000226037700045.

11. Hanage WP, Auranen K, Syrjanen R, Herva E, Makela PH, Kilpi T, et al. Ability of pneumococcal serotypes and clones to cause acute otitis media: Implications for the prevention of otitis media by conjugate vaccines. Infection and Immunity. 2004;72(1):76-81. doi: 10.1128/iai.72.1.76-81.2004. PubMed PMID: WOS:000187631600010.

12. Parra EL, Ramos V, Sanabria O, Moreno J. Serotype and Genotype Distribution among Invasive Streptococcus pneumoniae Isolates in Colombia, 2005-2010. Plos One. 2014;9(1). doi: 10.1371/journal.pone.0084993. PubMed PMID: WOS:000329862500203.

13. Pichon B, Ladhani SN, Slack MPE, Segonds-Pichon A, Andrews NJ, Waight PA, et al. Changes in Molecular Epidemiology of Streptococcus pneumoniae Causing Meningitis following Introduction of Pneumococcal Conjugate Vaccination in England and Wales. Journal of Clinical Microbiology. 2013;51(3):820-7. doi: 10.1128/jcm.01917-12. PubMed PMID: WOS:000315121700013.

14. Tanaka J, Ishiwada N, Wada A, Chang B, Hishiki H, Kurosaki T, et al. Incidence of childhood pneumonia and serotype and sequence-type distribution in Streptococcus pneumoniae isolates in Japan. Epidemiology and Infection. 2012;140(6):1111-21. doi: 10.1017/s0950268811001592. PubMed PMID: WOS:000304007700018.

15. Hsieh YC, Huang YC, Lin HC, Ho YH, Chang KY, Huang LM, et al. Characterization of invasive isolates of Streptococcus pneumoniae among Taiwanese children. Clinical Microbiology and Infection. 2009;15(11):991-6. doi: 10.1111/j.1469-0691.2009.02743.x. PubMed PMID: WOS:000271055600007.

16. Munoz R, Musser JM, Crain M, Briles DE, Marton A, Parkinson AJ, et al. GEOGRAPHIC-DISTRIBUTION OF PENICILLIN-RESISTANT CLONES OF STREPTOCOCCUS-PNEUMONIAE - CHARACTERIZATION BY PENICILLIN-BINDING PROTEIN PROFILE, SURFACE PROTEIN-A TYPING, AND MULTILOCUS ENZYME ANALYSIS. Clinical Infectious Diseases. 1992;15(1):112-8. PubMed PMID: WOS:A1992JA30000017.

17. Buckee CO, Jolley KA, Recker M, Penman B, Kriz P, Gupta S, et al. Role of selection in the emergence of lineages and the evolution of virulence in Neisseria meningitidis. Proceedings of the National Academy of Sciences of the United States of America. 2008;105(39):15082-7. doi: 10.1073/pnas.0712019105. PubMed PMID: WOS:000261914300046.

18. Watkins ER, Maiden MCJ. Persistence of Hyperinvasive Meningococcal Strain Types during Global Spread as Recorded in the PubMLST Database. Plos One. 2012;7(9). doi: 10.1371/journal.pone.0045349. PubMed PMID: WOS:000309973900038.

19. Brehony C, Wilson DJ, Maiden MCJ. Variation of the factor H-binding protein of Neisseria meningitidis. Microbiology-Sgm. 2009;155:4155-69. doi: 10.1099/mic.0.027995-0. PubMed PMID: WOS:000272918200035.

20. Callaghan MJ, Jolley KA, Maiden MCJ. Opacity-associated adhesin repertoire in hyperinvasive Neisseria meningitidis. Infection and Immunity. 2006;74(9):5085-94. doi: 10.1128/iai.00293-06. PubMed PMID: WOS:000240296400014.

21. Murphy E, Lin SL, Nunez L, Andrew L, Fink PS, Dilts DA, et al. Challenges for the evaluation of Staphylococcus aureus protein based vaccines Monitoring antigenic diversity. Human Vaccines. 2011;7:51-9. doi: 10.4161/hv.7.0.14562. PubMed PMID: WOS:000288984200009.

22. Tavares A, Faria NA, de Lencastre H, Miragaia M. Population structure of methicillin-susceptible Staphylococcus aureus (MSSA) in Portugal over a 19-year period (1992-2011). European Journal of Clinical Microbiology & Infectious Diseases. 2014;33(3):423-32. doi: 10.1007/s10096-013-1972-z. PubMed PMID: WOS:000331709700017.

23. McGregor KF, Spratt BG, Kalia A, Bennett A, Bilek N, Beall B, et al. Multilocus sequence typing of Streptococcus pyogenes representing most known emm types and distinctions among subpopulation genetic structures. Journal of Bacteriology. 2004;186(13):4285-94. doi: 10.1128/jb.186.13.4285-4294.2004. PubMed PMID: WOS:000222189500026.

24. Enright MC, Spratt BG, Kalia A, Cross JH, Bessen DE. Multilocus sequence typing of Streptococcus pyogenes and the relationships between emm type and clone. Infection and Immunity. 2001;69(4):2416-27. doi: 10.1128/iai.69.4.2416-2427.2001. PubMed PMID: WOS:000167616500055.

25. Achtman M, Wain J, Weill F-X, Nair S, Zhou Z, Sangal V, et al. Multilocus Sequence Typing as a Replacement for Serotyping in Salmonella enterica. Plos Pathogens. 2012;8(6). doi: 10.1371/journal.ppat.1002776. PubMed PMID: WOS:000305987800043.

26. Musser JM, Kroll JS, Moxon ER, Selander RK. EVOLUTIONARY GENETICS OF THE ENCAPSULATED STRAINS OF HEMOPHILUS-INFLUENZAE. Proceedings of the National Academy of Sciences of the United States of America. 1988;85(20):7758-62. doi: 10.1073/pnas.85.20.7758. PubMed PMID: WOS:A1988Q580700071.

27. Meats E, Feil EJ, Stringer S, Cody AJ, Goldstein R, Kroll JS, et al. Characterization of encapsulated and noncapsulated Haemophilus influenzae and determination of phylogenetic relationships by multilocus sequence typing. Journal of Clinical Microbiology. 2003;41(4):1623-36. doi: 10.1128/jcm.41.4.1623-1636.2003. PubMed PMID: WOS:000182179900042.

28. Duncan SS, Valk PL, Shaffer CL, Bordenstein SR, Cover TL. J-Western Forms of Helicobacter pylori cagA Constitute a Distinct Phylogenetic Group with a Widespread Geographic Distribution. Journal of Bacteriology. 2012;194(6):1593-604. doi: 10.1128/jb.06340-11. PubMed PMID: WOS:000300846900032.

29. Maslow JN, Whittam TS, Gilks CF, Wilson RA, Mulligan ME, Adams KS, et al. CLONAL RELATIONSHIPS AMONG BLOOD-STREAM ISOLATES OF ESCHERICHIA-COLI. Infection and Immunity. 1995;63(7):2409-17. PubMed PMID: WOS:A1995RE84200004.

30. Killgore G, Thompson A, Johnson S, Brazier J, Kuijper E, Pepin J, et al. Comparison of seven techniques for typing international epidemic strains of Clostfidium difficile: Restriction endonuclease analysis, pulsed-field gel electrophoresis, PCR-ribotyping, multilocus sequence typing, multilocus variable-number tandem-repeat analysis, amplified fragment length polymorphism, and surface layer protein A gene sequence typing. Journal of Clinical Microbiology. 2008;46(2):431-7. doi: 10.1128/jcm.01484-07. PubMed PMID: WOS:000253100300005.

31. Piffaretti JC, Kressebuch H, Aeschbacher M, Bille J, Bannerman E, Musser JM, et al. GENETIC-CHARACTERIZATION OF CLONES OF THE BACTERIUM LISTERIA-MONOCYTOGENES CAUSING EPIDEMIC DISEASE. Proceedings of the National Academy of Sciences of the United States of America. 1989;86(10):3818-22. doi: 10.1073/pnas.86.10.3818. PubMed PMID: WOS:A1989U652300081.

32. Bilek N, Ison CA, Spratt BG. Relative Contributions of Recombination and Mutation to the Diversification of the opa Gene Repertoire of Neisseria gonorrhoeae. Journal of Bacteriology. 2009;191(6):1878-90. doi: 10.1128/jb.01518-08. PubMed PMID: WOS:000263819500016.

33. Vimont S, Mnif B, Fevre C, Brisse S. Comparison of PFGE and multilocus sequence typing for analysis of Klebsiella pneumoniae isolates. Journal of Medical Microbiology. 2008;57(10):1308-10. doi: 10.1099/jmm.0.2008/003798-0. PubMed PMID: WOS:000259950400023.

34. Cody AJ, Maiden MJC, Dingle KE. Genetic diversity and stability of the porA allele as a genetic marker in human Campylobacter infection. Microbiology-Sgm. 2009;155:4145-54. doi: 10.1099/mic.0.031047-0. PubMed PMID: WOS:000272918200034.

35. Bennett JS, Thompson EAL, Kriz P, Jolley KA, Maiden MCJ. A common gene pool for the Neisseria FetA antigen. International Journal of Medical Microbiology. 2009;299(2):133-9. doi: 10.1016/j.ijmm.2008.06.010. PubMed PMID: WOS:000263788000005.

36. Chowdhury SA, Arias CA, Nallapareddy SR, Reyes J, Willems RJL, Murray BE. A Trilocus Sequence Typing Scheme for Hospital Epidemiology and Subspecies Differentiation of an Important Nosocomial Pathogen, Enterococcus faecalis. Journal of Clinical Microbiology. 2009;47(9):2713-9. doi: 10.1128/jcm.00667-09. PubMed PMID: WOS:000269439600004.

37. Ahmad Y, Gertz RE, Jr., Li Z, Sakota V, Broyles LN, Van Beneden C, et al. Genetic Relationships Deduced from emm and Multilocus Sequence Typing of Invasive Streptococcus dysgalactiae subsp equisimilis and S-canis Recovered from Isolates Collected in the United States. Journal of Clinical Microbiology. 2009;47(7):2046-54. doi: 10.1128/jcm.00246-09. PubMed PMID: WOS:000267713000009.

38. Davies RL, Arkinsaw S, Selander RK. Genetic relationships among Pasteurella trehalosi isolates based on multilocus enzyme electrophoresis. Microbiology-Uk. 1997;143:2841-9. PubMed PMID: WOS:A1997XQ87500036.

39. Frandsen EVG, Wade WG. Differentiation of human Capnocytophaga species by multilocus enzyme electrophoretic analysis and serotyping of immunoglobulin A1 proteases. Microbiology-Uk. 1996;142:441-8. PubMed PMID: WOS:A1996TW47700025.

40. Kucharski AJ, Andreasen V, Gog, JR.Capturing the dynamics of pathogens with many strains. Journal of Mathematical Biology 2015; 03/2015; DOI:10.1007/s00285-015-0873-4

41. Gupta S, Ferguson N, Anderson R. Chaos, persistence, and evolution of strain structure in antigenically diverse infectious agents. Science. 1998;280(5365):912-5. doi: 10.1126/science.280.5365.912. PubMed PMID: WOS:000073532900044.

42. Feikin DR, Klugman KP. Historical changes in pneumococcal serogroup distribution: Implications for the era of pneumococcal conjugate vaccines. Clinical Infectious Diseases. 2002;35(5):547-55. doi: 10.1086/341896. PubMed PMID: WOS:000177431000007
